# *Chaetomorpha linum* polysaccharides alleviate NAFLD in mice by enhancing the PPARα/CPT-1/MCAD signaling

**DOI:** 10.1186/s12944-022-01730-x

**Published:** 2022-12-19

**Authors:** Xueru Chu, Yu Zhou, Shuimi Zhang, Shousheng Liu, Guoyun Li, Yongning Xin

**Affiliations:** 1 School of Medicine and Pharmacy, Ocean University of China, Department of Infectious Disease, Qingdao Municipal Hospital, 5 Yushan Road, Qingdao, 266003, 266011 Shandong Province China; 2 Clinical Research Center, Qingdao Municipal Hospital, Qingdao, 266071 Shandong Province China; 3 Department of Infectious Disease, Qingdao Municipal Hospital, 1 Jiaozhou Road, Qingdao, 266011 Shandong Province China

**Keywords:** Nonalcoholic fatty liver disease, Chaetomorpha linum polysaccharide, Arabinogalactan, Glucose tolerance, Mice

## Abstract

**Background:**

Green algae contain many polysaccharides. However, there is no information on whether *Chaetomorpha linum* polysaccharides (CLP) can modulate lipid and glucose metabolism.

**Material and methods:**

CLP were extracted from chlorella and their components were characterized. Male C57BL/6 mice were randomized and provided with control chow as the control, or high fat diet (HFD) to induce nonalcoholic fatty liver disease (NAFLD). NAFLD mice were treated orally with water as the HFD group or with 50 or 150 mg/kg CLP daily for 10 weeks. The impact of CLP treatment on lipid and glucose metabolism and the PPARα signaling was examined by histology, Western blotting and biochemistry.

**Results:**

CLP mainly contained arabinogalactan sulfate. Compared with the control, HFD feeding increased body weights, lipid droplet liver deposition and induced hyperlipidemia, liver functional impairment and glucose intolerance in mice. Treatment with CLP, particularly with a higher dose of CLP, limited the HFD-increased body weights and liver lipid droplet deposition, mitigated the HFD-induced hyperlipidemia and improved liver function and glucose tolerance in mice. Mechanistically, feeding with HFD dramatically decreased the expression of liver PPARα, CPT-1, and MCAD, but treatment with CLP enhanced their expression in a trend of dose-dependent in mice.

**Conclusions:**

These findings indicated that CLP treatment alleviated the gain in body weights, NAFLD, and glucose intolerance in mice after HFD feeding by enhancing the PPARα/CPT-1/MCAD signaling.

## Introduction

Nonalcoholic fatty liver disease (NAFLD) is a common chronic liver disease with the characterization of excess fat accumulation in the liver, which may cause liver functional impairment and tissue damage [1, 2]. Although early NAFLD does not have clinical symptom and without appropriate intervention, it may lead to steatohepatitis, liver fibrosis, cirrhosis and liver cancer [3]. Accumulated evidence has indicated that dyslipidemia, insulin resistance, obesity, and impaired glucose tolerance are crucial for the development of NAFLD [4]. Currently, therapeutic strategies for early NAFLD include life-style changes, enhanced exercise and reduced body weights. However, there is no effective therapeutic medicine available for the cure of NAFLD in the clinic [5]. Hence, identification of new therapeutic agents that control dyslipidemia, obesity and glucose intolerance will be beneficial to NAFLD patients.

The peroxisome proliferator-activated receptor α (PPARα)-related signaling is important for liver lipid metabolism [6]. PPARα is a nuclear receptor of hormones and activated PPARα functions as a transcription factor to regulate the expression of its target genes and lipid metabolism, such as fatty acid *β*-oxidation [7]. Activated PPARα can promote the expression of carnitine palmitoyltransferase-1 (CPT-1) and medium-chain acyl-CoA dehydrogenase (MCAD), both of which are critical for the process of fatty acid *β*-oxidation [8, 9]. The CPT-1 can mediate the transport of long-chain fatty acids into mitochondria, where they are catalyzed by MCAD for *β*-oxidation [10]. Conceivably, activation of the PPARα-related signaling can promote fatty acid *β*-oxidation and degradation, alleviating hyperlipidemia and NAFLD.

A previous study has shown that pectin pollen polysaccharides from rose contain arabinogalactan domain and can significantly enhance insulin sensitivity, glucose metabolism and reduce hepatic steatosis in obese mice [11]. The water-soluble polysaccharides, JS-MP-1, from Mulberry fruit in Korea mainly contain arabinogalactan side chain and can inhibit the proliferation of preadipose cells to reduce the mass of adipose tissues [12]. Chlorella belongs to chlorophyta, and is an unbranched filamentous single-celled green algae growing in estuaries and the marine environments [13]. There are about 76 species of trichomes [14] and among them, flax plants have high humidity, and the dried residues are mainly composed of proteins and carbohydrates. Chlorella is mainly used as an ecological regulator of the nutrient availability of estuarine habitats. Chlorella is widely present in China and has been used as a dietary fiber and medicine [15]. *Chaetomorpha linum* is a wild chlorella species and grown along the shores of Swan Lake in Rongcheng, China. However, whether treatment with *Chaetomorpha linum* polysaccharides (CLP) can modulate liver lipid metabolism, glucose intolerance and NAFLD has not been investigated.

This study aimed at extracting and characterizing CLP, and investigating the therapeutic impact of CLP treatment on NAFLD and the pharmacological mechanisms in NAFLD mice following high fat diet (HFD) feeding.

## Materials and methods

### Preparation and chemical characterization of CLP

Wild *Chaetomorpha linum* was obtained from the shores of Swan Lake in Rongcheng, China, dried at 55 °C and pulverized. To remove lipids, the pulverized algae was extracted with 85% ethanol (1: 20 weight: volume) for 3 h (three times) at 80 °C. After being dried at 55 °C, the defatted residues were extracted with room temperature water at 1: 45 for 3 h (3 times), centrifuged and further extracted with hot water (at 1: 45) for 3 h (3 times) at 85 °C, followed by filtration. The extracts were concentrated by rotary evaporation and precipitated with ethanol. The precipitates were dissolved in water, dialyzed (7000 Da MWCO) against water for 3 days, concentrated and lyophilized as the CLP. The contents of carbohydrates in the CLP were quantified by a phenol–sulfuric acid assay using arabinose as a standard. The levels of sulfate in CLP were quantified using the barium chloride-gelatin assay [16], while the levels of proteins in CLP were measured using the Folin-phenol reagent [17]. The CLP were also characterized for their purity and average molecular weight (Mw) by HPGPC-MALLS [18] using two columns (HQSB 804 and HQSB 802.5), 0.1 Na_2_SO_4_ elution for mobile phase with a flow rate of 0.6 mL/min 35 C and multi-angle laser light scattering. The composition of CLP was characterized by PMP-HPLC with a ZORBAX Eclipse XDB-C18 column (4.6 mm, 150 mm, 5 m).

### Animals and experimental design

C57BL/6 J mice (male, 7 weeks in age) were obtained from the Beijing Vital River (China) and raised in a specific pathogen-free room with a light–dark cycle of 12 h at 23 °C-25°C and free-access to food and water ad libitum. The mice were randomized and provided with standard rodent chow (D12450B, 3.85 kcal/g, 10% calories from fat, Research Diets, New Brunswick, NJ, USA) as the control, or with HFD (D12492, 5.24 total kcal/g, 60% calories from fat, Research Diets) for 10 weeks. The mice with HFD were separated and fed with vehicle water as the HFD group or CLP in water at 50 mg/kg/day (the CLP-50 group) or 150 mg/kg/day (the CLP-150 group) daily by gavage for 10 weeks. After being food-fasted overnight, animals were anesthetized with sodium pentobarbital (100 mg/kg, intraperitoneal injection). Their peripheral blood samples were obtained for the preparation of serum samples. The mice were humanly euthanized and their liver tissues were dissected. The Animal Experiment and Care Committee of Ocean University of China approved all animal experiments.

### Determination of liver histopathology, serum and hepatic lipid profiles

Fresh liver tissues were washed with phosphate buffer solution (PBS) and weighed. Partial liver tissue Sects. (4 µm) were routine-stained with hematoxylin and eosin (H&E) or oil red O. The sections were examined and photoimaged using a light microscope (Olympus, Japan). The remaining liver tissues were stored at -80 °C until use. Their serum triglyceride (TG), total cholesterol (TC), high-density lipoprotein (HDL), low-density lipoprotein (LDL) concentrations were measured for lipid profiles, and aspartate aminotransferase (AST), alanine aminotransferase (ALT) were quantified for liver functional measures as well as fasting blood glucose (FBG) were tested. All the above serum indicators were tested with the commercial kits (Nanjing Jiancheng Institute of Bioengineering, China) following the manufacturer's instructions. Individual liver sample (10 mg each) was homogenized in 90 μl of anhydrous ethanol in a PotterElvehjem tissue homogenizer and centrifuged at 2500 × g for 10 min to obtain liver tissue extract. The concentrations of TG and TC in the liver were measured using the commercial kits (Nanjing Jiancheng Institute of Bioengineering), following the manufacturer's instructions.

### Oral glucose tolerance test (OGTT)

One day before the mice were euthanized, they were fasted for 6 h and their glucose tolerance was analyzed by OGTT. In brief, individual mice were administrated with D-glucose (2 g/kg body weight) by gavage and their blood glucose concentrations were tested before and 30, 60, and 120 min after glucose challenge with a glucometer (OMRON, JAPAN).

### Quantitative real-time polymerase chain reaction (qRT-PCR)

Total RNA was extracted from individual liver tissue samples using Trizol (RNAex Pro Reagent, AG). The RNA samples (1 μg each) were reversely transcribed into cDNA with the Primescript MRT kit (Takara, Kyoto, Japan), respectively. The interested gene mRNA transcripts relative to the control *β*-actin in each liver tissue was quantified in triplicate by qRT-PCR using the SYBR Green PCR kit (Qiagen, Dusseldorf, Germany) and specific primers (Table 1). The data were analyzed by 2^−ΔΔCt^ method.

**Table 1:** The sequences of primers

Primer	Forward sequence	Reverse sequence
PPAR-α	GGAGTGCAGCCTCAGCCAAGTT	AGGCCACAGAGCGCTAAGCTGT
CPT-1	AAGAACATCGTGAGTGGCGTC	AGCACCTTCAGCGAGTAGCG
MCAD	AGAGGAGATTATCCCCGTCGC	TACGCCAACTCTTCGGTAATTAAAC

### Western blotting

The impact of CLP treatment on the expression and phosphorylation of relevant proteins in liver tissues was examined by Western blotting. In briefly, each liver tissue sample was homogenized in RIPA buffer reagent containing protease and phosphatase inhibitors (Qiagen). The tissue lysates (30 µg/lane) were subjected to sodium dodecyl-sulfate polyacrylamide gel electrophoresis on 10% gels and transferred onto nitrocellulose membranes (Bio-Rad, USA). After being blocked with 5% skimmed dry milk in TBST buffer, the membranes were reacted with anti-PPAR-α (D161086, Sangon, China), anti-MCAD (GB112107, Servicebio, China), anti-CPT-1 (sc-393070), and GAPDH (sc-47724, Santa Cruz Biotech, USA) overnight at 4 oC. After being washed, the bound antibodies were reacted with horseradish peroxidase (HRP)-conjugated secondary antibodies (Santa Cruz Biotechnology) and observed with enhanced chemiluminescent plus reagents (Amersham Biosciences, USA). The data were quantitatively analyzed using the Image-Pro Plus software.

### Statistical analysis

Data are presented as the mean ± standard error mean (SEM). The comparison between groups was performed by one-way ANOVA and post hoc Student's t test and the multiple comparisons were analyzed by Tukey's honestly significant difference test using SPSS software (version 20.0; IBM, Armonk, NY, USA). The statistically significant difference between various groups was considered when a *P*-value of < 0.05.

## Results

### Chemical characterization of CLP

**Fig. 1:**
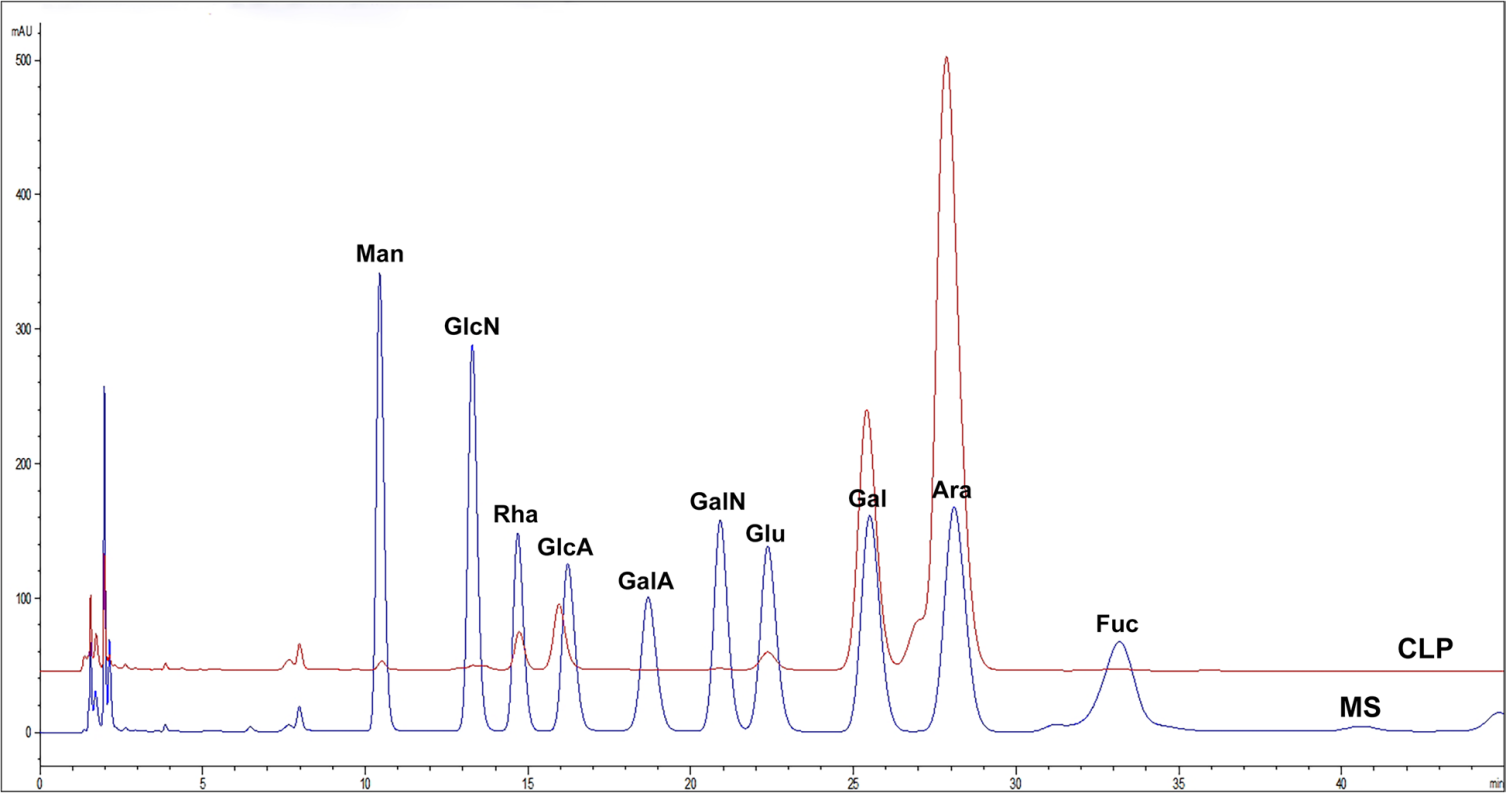
HPLC analysis of CLP monosaccharide components. CLP, *Chaetomorpha linum* polysaccharide; MS, monosaccharide standards. Man, mannose; GlcN, glucosamine; Rha, rhamnose; GlcA, glucuronic acid; GalA, galactonic acid; GalN, galactosamine; Glc, glucose; Gal, galactose; Ara, arabinose; Fuc, fructose

CLP were characterized for their purity and average molecular weight (Mw) by HPGPC-MALLS. As the Fig. 1 shown, the peak shape of CLP is mainly a single peak, and the distribution range is narrow, indicating that the molecular weight distribution of CLP is more concentrated. Polysaccharide showed a symmetric peak on a PL aquagel OH column, with average molecular weight of 521.1 kDa. HPLC monosaccharide composition analysis indicated that CLP consisted of arabinose (Ara), galactose (Gal), a small amount of rhamnose (Rha), glucose (Glu) and glucuronic acid (GlcA) (Fig. 1 and Table 2). Theses results suggested that CLP is not highly pure polysaccharides, CLP mainly contained arabinogalactan sulfate, but also other components. In addition, the total sugar content, protein content and sulfate content of CLP were determined. With arabinose as the standard, the total sugar content of CLP was 77.7%, the protein content of CLP was 15.4%, and the sulfate content of CLP was 1.6%.

**Table 2:** Analysis of CLP monosaccharide compositions

	Rha	GlcA	Glu	Gal	Xyl	Ara
CLP	3.7	6.8	1.8	21.5	—	66.2

### CLP treatment reduces body weights in the mice receiving HFD

**Fig. 2:**
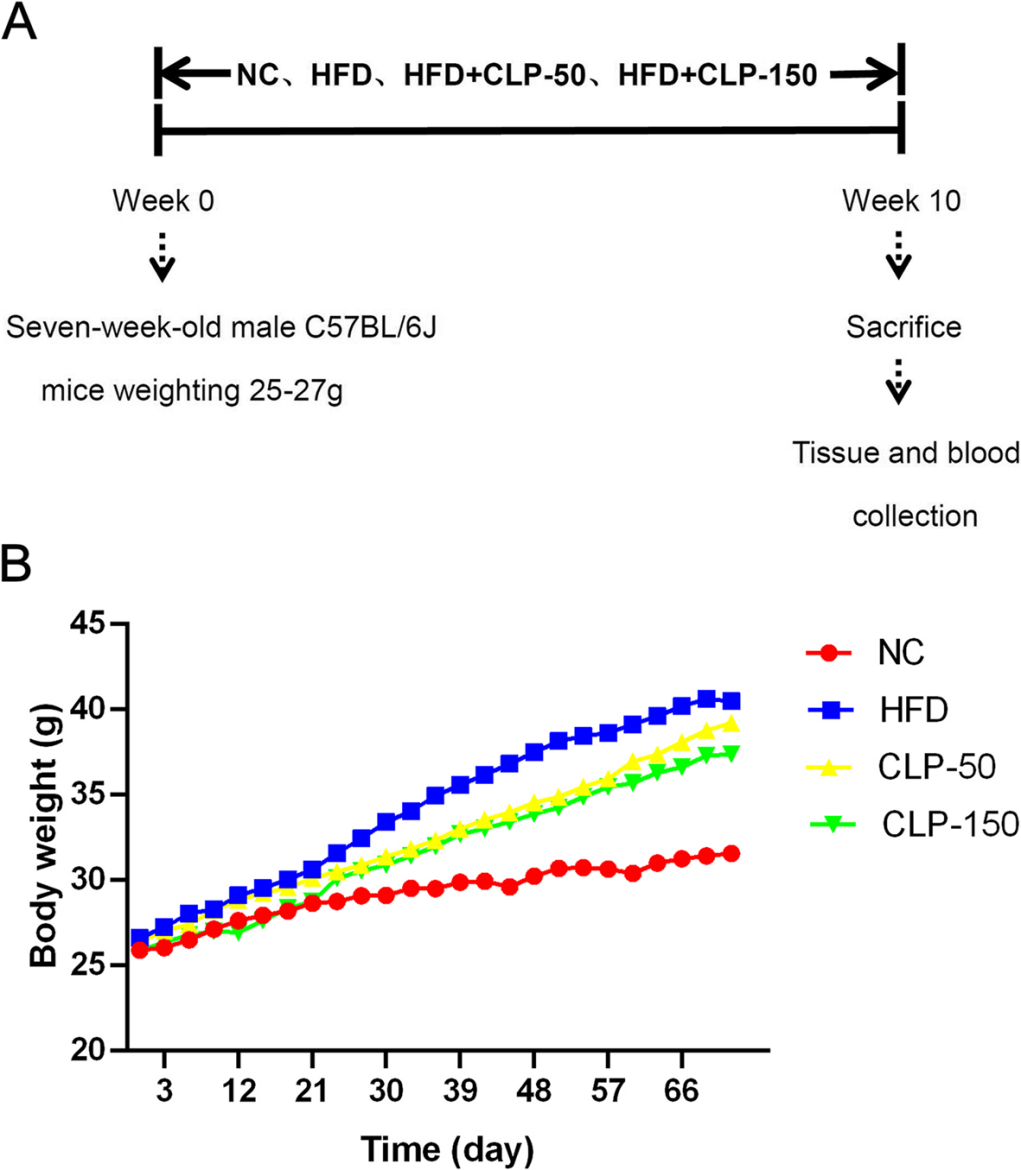
CLP treatment mitigates the gain in body weights of mice. **A** A schematic diagram for experimental design. **B** Longitudinal measurements of body weights in different groups of mice. Data are the mean body weights of each group (*n* = 6 per group). The NC group, HFD group, CLP-50 group, and CLP-150 group. NC, control; HFD, HFD-fed mice; CLP-50, HFD with 50 mg/kg/d of CLP; CLP-150, HFD with 150 mg/kg/d of CLP

To determine the potential impact of CLP treatment, mice were fed with HFD to establish NAFLD and treated with varying doses of CLP for 10 weeks (Fig. 2A). First, comparable amounts of daily food and water consumption were observed in those different groups of mice (data not shown). Longitudinal measurements of their body weights displayed that relative to the NC group, feeding with HFD for 10 weeks moderately increased body weights in mice (*P* < 0.05 for all) and treatment with both CLP-50 and CLP-150 significantly reduced the gain in body weights (*P* < 0.05 for all, Fig. 2B). Hence, CLP treatment significantly mitigated the gain in body weights in the mice following feeding with HFD.

### CLP treatment decreases fat deposition in the liver of mice with HFD feeding

**Fig. 3:**
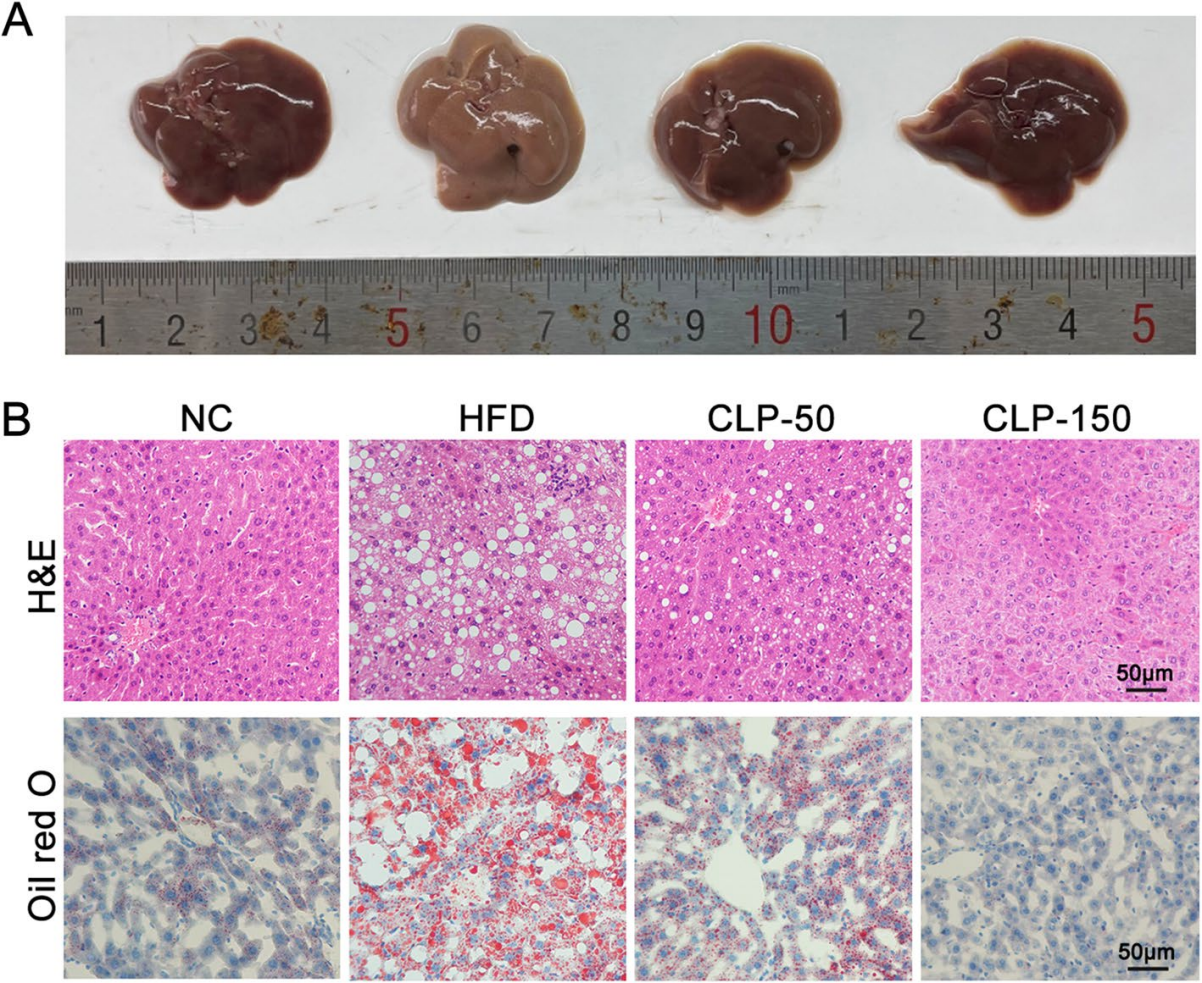
CLP treatment limits the lipid droplet deposition in the liver of mice. **A** The representative gross liver tissues from the indicated groups of mice at the end of the experiment. **B** H&E and oil red O staining of liver sections from the indicated groups of mice. NC, the control group; HFD, the HFD-fed mice receiving vehicle treatment; CLP-50, the HFD-fed mice receiving 50 mg/kg/d CLP; CLP-150, The HFD-fed mice receiving 150 mg/kg/d CLP

While the gross liver tissues from the NC group of mice displayed brown color the gross liver tissues from the HFD group exhibited obvious white and abundant lipid droplets, an indicative of lipid liver deposition (Fig. 3). However, there were a few white lipid droplets in the CLP-treated mice with HFD feeding, particularly for those receiving a higher dose of CLP. Histologically, there were many white larger lipid droplets in the liver of the HFD group of mice (Fig. 3B). In contrast, there were a few smaller lipid droplets in the liver of the CLP-50 group of mice and no obvious lipid droplet in the liver of the CLP-150 group of mice. The oil red O staining revealed that there was little in the livers of the NC and CLP-150 groups of mice while extensive red staining of triglycerides and lipids was observed in the liver of mice in the HFD group. In contrast, obviously less staining was detected in the liver of the CLP50 group of mice. Together, the results indicated that CLP treatment mitigated the lipid liver deposition in the mice with HFD feeding in a trend of dose-dependent.

### Treatment with a higher dose of CLP prevents hyperlipidemia and improves liver function in mice with HFD feeding

**Fig. 4:**
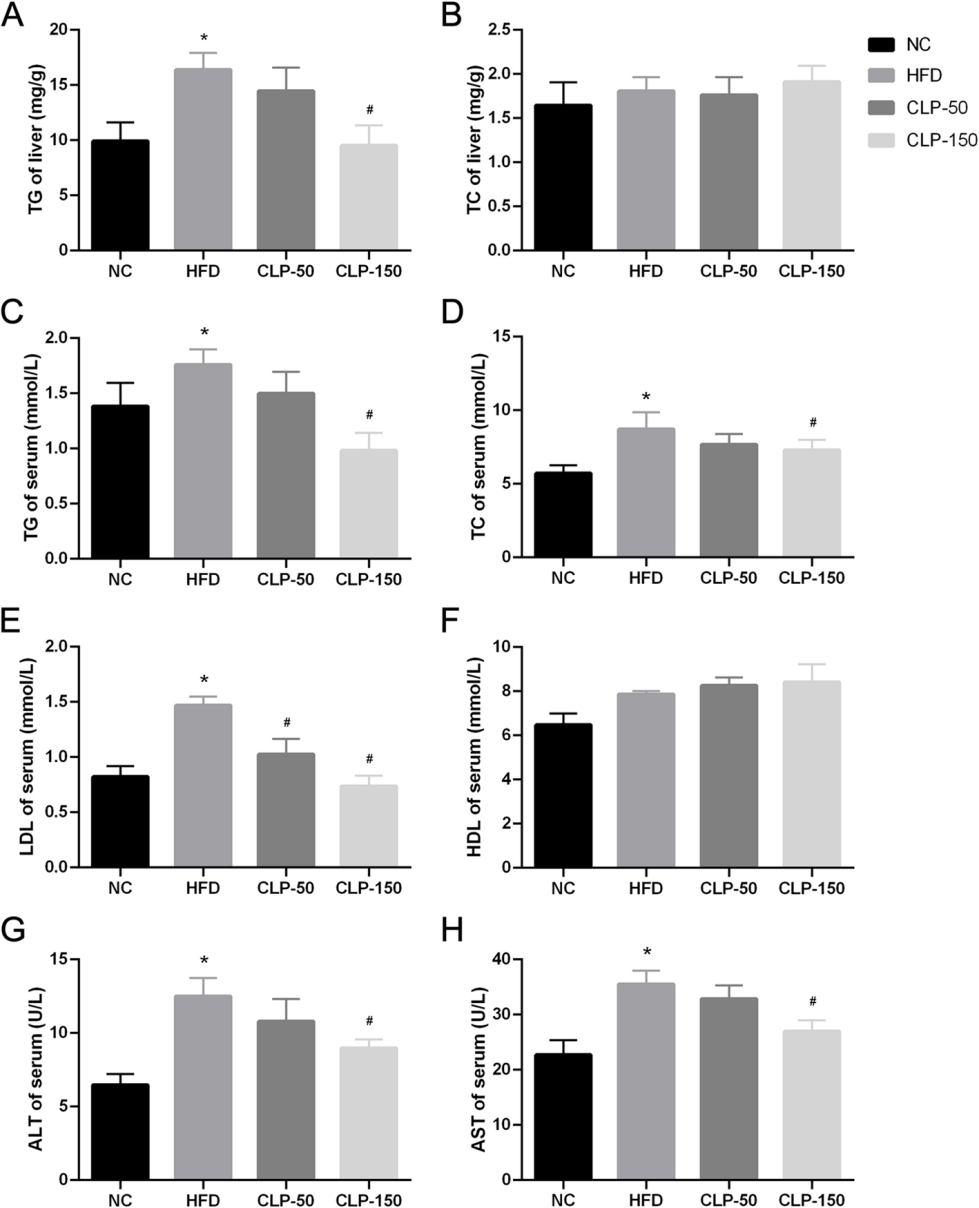
Quantitative analyses of liver and serum lipids and liver functional enzymes in mice. **A** The levels of hepatic TG in the indicated groups of mice; **B** The levels of hepatic TC in the indicated groups of mice. **C-F** The levels of serum TG **C**, TC **D**, LDL **E**, and HDL **F** in the indicated groups of mice. **G-H** The levels of serum ALT **G** and AST **H** in the indicated groups of mice. NC: The control group; HFD: The HFD-fed mice receiving vehicle treatment; CLP-50: The HFD-fed mice receiving 50 mg/kg/d CLP; CLP-150: The HFD-fed mice receiving 150 mg/kg/d CLP. Data were analyzed by student's t test and expressed as the mean ± SEM. ^*^P < 0.05 vs. the NC group; ^#^ *P* < 0.05 vs. the HFD group

Analysis of hepatic TG and TC unveiled that compared with the NC control, higher levels of hepatic TG were detected in the HFD group (*P* < 0.05), which was significantly abrogated in the CLP-150-treated mice, but not in the CLP-50-treated mice (*P* < 0.05, Fig. 4A). The concentrations of hepatic TC were comparable among those groups of mice (all *P* > 0.05, Fig. 4B). Measurements of serum lipids displayed that feeding with HFD for 10 weeks significantly increased serum TG, TC and LDL levels, relative to that in the control mice (all *P* < 0.05, Fig. 4C-E). In comparison with the HFD group, treatment with a lower dose of CLP significantly mitigated the gain in serum LDL levels (*P* < 0.05), but only slightly decreased serum TG and TC contents in mice (*P* > 0.05). However, treatment with a higher dose of CLP significantly prevented the HFD-increased serum TG and LDL levels and reduced the gain in serum TC in mice (*P* < 0.05 for all). The concentrations of serum HDL were similar among those different groups of mice (*P* > 0.05, Fig. 4F). Analysis of serum ALT and AST concentrations revealed that relative to the control group, HFD-feeding significantly elevated serum ALT and AST concentrations in mice, which were slightly reduced in the CLP-50-treated mice (*P* > 0.05 for all), but significantly mitigated in the CLP-150-treated mice (*P* < 0.05 for all, Figs. 4G and H). Thus, the results unveiled that treatment with a higher dose of CLP prevented hyperlipidemia and improved liver function in mice with HFD feeding.

### CLP treatment improves glucose intolerance in mice fed with HFD

**Fig. 5:**
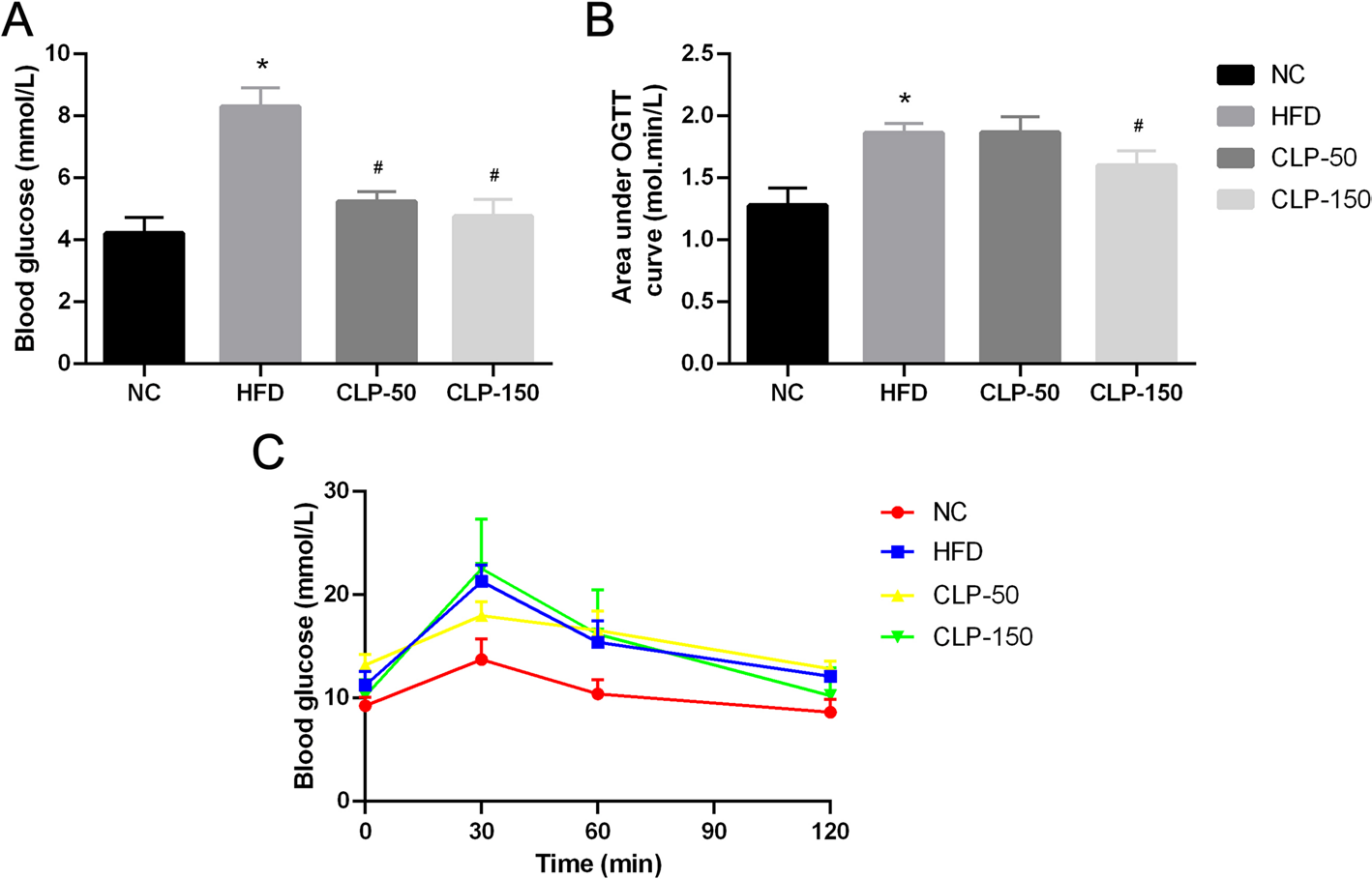
CLP treatment improves glucose intolerance in the HFD-fed mice. After fasted for 6 h, individual mice were administrated with D-glucose (2 g/kg body weight) by gavage and their blood glucose levels were measured before as the fasting blood glucose and 30, 60, 90, and 120 min post glucose injection. **A** The levels of fasting blood glucose. **B** The dynamic changes in blood glucose levels in individual groups of mice. **C** The area under curve of OGTT. NC: The control; HFD: The HFD-fed mice receiving vehicle treatment. CLP-50: The HFD-fed mice receiving 50 mg/kg/d CLP; CLP-150: The HFD-fed mice receiving 150 mg/kg/d CLP. Data were analyzed by student’s t test and expressed as the mean ± SEM.^*^P < 0.05 vs. the NC group; ^#^*P* < 0.05 vs. the HFD group

Next, the impact of CLP treatment on glucose tolerance was tested by OGTT. Compared with the control group, the concentrations of FBG were significantly elevated in the HFD group of mice (*P* < 0.05, Fig. 5A). Treatment with either dose of CLP significantly reduced FBG concentrations in mice with HFD feeding (*P* < 0.05 for all). OGTT displayed that following oral administration of glucose the concentrations of blood glucose in the mice with HFD feeding were higher than in the control mice (Fig. 5B). While blood glucose levels in the control and CLP-150-treated groups decreased to 10 mM the blood glucose levels in the HFD and CLP-50 groups remained significantly higher than 10 mM. As a result, the AUC values of dynamic blood glucose levels in the HFD and CLP-50 groups were significantly higher than that in the control and CLP-150 groups (*P* < 0.05 for all, Fig. 5C). Accordingly, treatment with CLP, particularly with a higher dose, significantly improved glucose intolerance in mice after feeding with HFD.

### CLP administration significantly enhances the PPARα signaling in the liver of mice

**Fig. 6:**
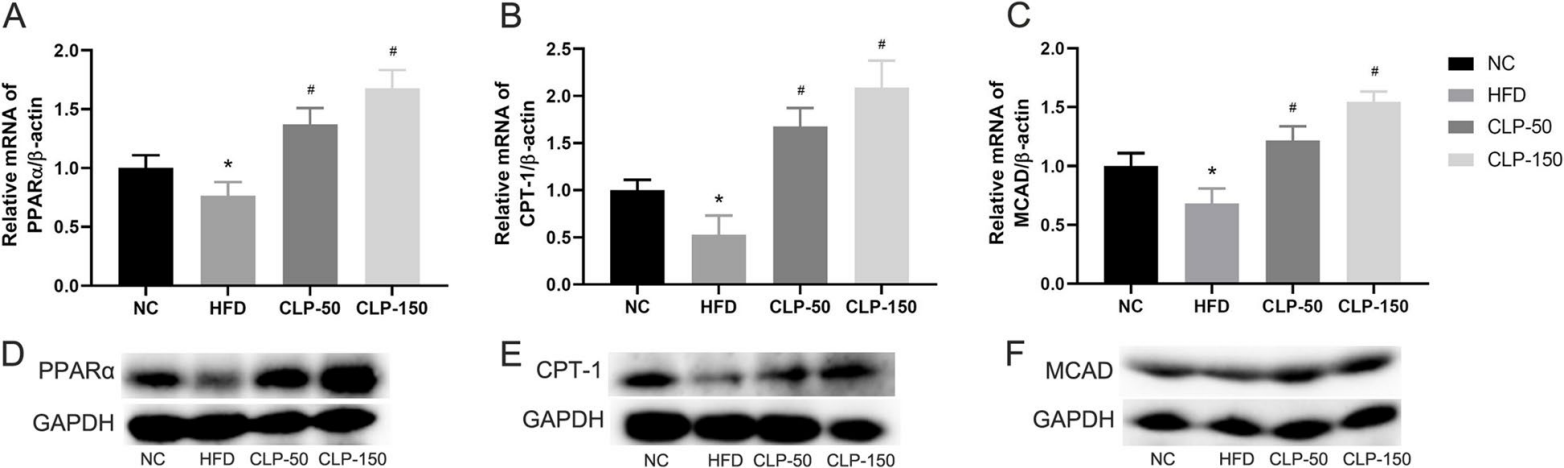
CLP treatment enhances the PPARα/CPT-1/MCAD signaling in the liver of HFD-fed mice. The relative levels of PPARα, CPT-1 and MCAD expression in the liver of individual mice were analyzed by qRT-PCR and Western blotting. **A-C** qRT-PCR analyses of the relative expressions of PPARα, CPT-1, and MCAD in the liver of the indicated groups of mice. **D-F** Western blotting analyses of the relative levels of PPARα, CPT-1, and MCAD in the liver of the indicated groups mice. NC: The control; HFD: The HFD-fed mice receiving vehicle treatment; CLP-50: The HFD-fed mice receiving 50 mg/kg/d CLP; CLP-150: The HFD-fed mice receiving 150 mg/kg/d CLP. Data were analyzed by student's t test and expressed as the mean ± SEM.^*^P < 0.05 vs. the NC group; ^#^*P* < 0.05 vs. the HFD group

The PPARα signaling is critical for liver metabolism. To understand the pharmacological action of CLP, the impact of CLP on the levels of hepatic PPARα, CPT-1, and MCAD expression in the different groups of mice was quantified by qRT-PCR and Western blotting. Relative to the control group, significantly lower PPARα, CPT-1, and MCAD expression was observed in the liver of the HFD group, but higher levels of them were detected in the CLP-treated groups of mice (*P* < 0.05 for all, Fig. 6). The effects of CLP treatment on PPARα, CPT-1, and MCAD expression in the liver displayed a trend of dose-dependent. Therefore, CLP treatment significantly enhanced the PPARα/CPT-1/MCAD signaling, ameliorating the HFD-induced NAFLD in mice.

## Discussion

**Fig. 7:**
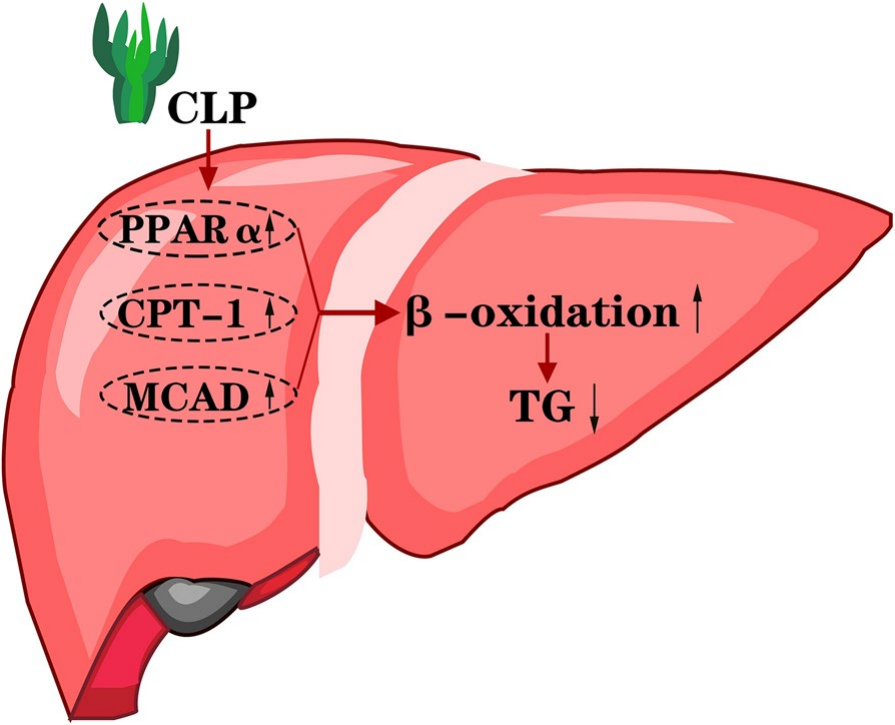
A proposed model of CLP alleviating NAFLD in the HFD-fed mice

NAFLD is the most prevalent public health problem worldwide because NAFLD is risk for the onset of heart diseases and type 2 diabetes [19, 20]. In recent years, the development of new therapeutic strategies for NAFLD becomes a hot field in scientific research. In this study, CLP was extracted from a wild chlorella species (*Chaetomorpha linum*) and mainly contained arabinogalactan sulfate. Functionally, CLP treatment significantly mitigated the body weight gain and limited the lipid droplet deposition in the liver of NAFLD mice. In addition, CLP treatment significantly modulated the systemic and liver lipid profiles, and enhanced glucose tolerance in mice with HFD feeding. Mechanistically, CLP administration enhanced the relative expression of hepatic PPARα, CPT-1 and MCAD in mice following HFD feeding. Conceivably, CLP administration significantly enhanced the PPARα/CPT-1/MCAD signaling to promote the degradation of fatty acids by β-oxidation in the liver, reducing the levels of systemic TG in the NAFLD mice after HFD induction (Fig. 7). These novel findings suggest that CLP may be valuable for the control of hyperlipidemia and may uncover new pharmacological mechanisms by which CLP administration ameliorates NAFLD.

Accumulated evidence has proven that plant polysaccharides and marine sulfate polysaccharides can regulate glycometabolism and metabolic syndrome, and these polysaccharides have been proposed as the potential therapeutic drugs for metabolism-related diseases [21–23]. The fucoidan from brown seaweeds can decrease the liver adipose droplet deposition and white adipose tissue weights, improving the serum lipid profiles, and hepatic steatosis in mice after feeding with HFD [24]. Similarly, carrageenans from *Sarconema filiforme* can limit an increase in the body weights, reduce body fat mass, and improve the serum lipid profiles in the HFD-fed rats, associated with modulating gut microbiota [25]. Moreover, the pectins containing the arabinogalactan domain obtained from the aqueous pulp extract of arecaceae can reduce the levels of TG and TC, and protect from liver injury in mice induced by hypercholesterolemic diet [26]. Moreover, arabinogalactan extracted from a kind of plants has potent anti-hyperglycemic effect by improving glucose intolerance and insulin resistance [11, 27, 28]. Similarly, treatment with a low molecular weight of fucoidan or high stability of fucoxanthin (LMF-HSFx) significantly reduces serum AST, ALT, TC, TG, and FBG levels and improves liver functional serum measures in NAFLD patients [29]. Oral fucoidan treatment significantly decreases the body fat mass, and improves the serum lipid profiles and hepatic steatosis in the ApoE (shl) mice following HFD feeding [29]. The results from the current study indicated that treatment with CLP that mainly contained arabinogalactan sulfate not only decreased the gain in body weights, hyperlipidemia, but also ameliorated glucose tolerance in the mice following HFD feeding, supporting the notion that therapy with the arabinogalactan-contained polysaccharides improves lipid and glucose metabolism, benefiting individuals with NAFLD and metabolic syndrome. Conceivably, the arabinogalactan-contained polysaccharides from other plants may also possess similarly beneficial effects.

The PPARα, γ, and β/δ act as receptors of fatty acids and their derivatives [30]. The PPARα is mainly expressed in the liver and other organs in the body and functions to control fatty acid degradation and glucose homeostasis by inducing the expression of downstream genes, such as the CPT-1 and MCAD [31, 32]. Actually, Saroglitazar, a novel PPARα agonist, has been demonstrated to ameliorate lipid and glucose metabolism in NAFLD patients [33–36]. Similarly, treatment with Silybin, another PPARα agonist, effectively alleviates the symptom of NAFLD in the clinic by reducing fat mass and inducing CPT-1 and MCAD expression [37]. Interestingly, treatment with CLP also enhanced the expression of hepatic PPARα, CPT-1 and MCAD in the HFD-fed mice, which may contribute to its beneficial effect on NAFLD. It is possible that CLP may alleviate NAFLD by enhancing the PPARα/CPT-1/MCAD signaling in the liver of mice after HFD induction. Given that there is no effective therapy for the control of NAFLD, the current findings suggest that CLP may be a potential therapeutic reagent for the effective intervention of NAFLD.

### Comparisons with other studies and what does the current work add to the existing knowledge

Available information has suggested that treatment with the arabinogalactan-contained polysaccharides from plants may improve lipid and glucose metabolism in animal models of NAFLD. However, there is no study using CLP. The findings from the current work included a) CLP from wild chlorella species (*Chaetomorpha linum*) contained mainly arabinogalactan sulfate; b) CLP treatment alleviated the symptoms of NAFLD, improved glucose intolerance and hyperlipidemia by enhancing the PPARα/CPT-1/MCAD signaling in mice after HFD induction. These novel findings extended previous observations and support the notion that arabinogalactan-contained polysaccharides ameliorate the development and progression of NAFLD.

### Study strengths and limitations

The strengths of this study were that the CLP was first isolated from wild chlorella species (*Chaetomorpha linum*) and its components were identified to contain mainly arabinogalactan sulfate. The findings also demonstrated that CLP treatment effectively ameliorated the progression of NAFLD by enhancing the PPARα/CPT-1/MCAD signaling in the liver of mice following HFD feeding. This study had several limitations. Firstly, the CLP mainly contained arabinogalactan sulfate, but also other components. The current study used a mixture of several components of CLP. Therefore, further studies should center on the effect of arabinogalactan sulfate in CLP. Secondly, the current study analyzed the expressions of PPARα/CPT-1/MCAD signaling events, but whether enhancing the PPARα/CPT-1/MCAD signaling can be the key factor for the CLP-mediated therapeutic effect remains to be determined.

## Conclusions

In summary, the CLP from a wild chlorella species (*Chaetomorpha linum*) contained mainly arabinogalactan sulfate. CLP treatment effectively mitigated an increase in body weights and the lipid droplet deposition in the liver of mice after HFD feeding. In addition, CLP administration improved hyperlipidemia and glucose tolerance in mice following HFD feeding. Mechanistically, CLP administration dramatically elevated the PPARα/CPT-1/MCAD signaling in the liver of mice following HFD feeding. The available findings indicated that CLP treatment ameliorated NAFLD and may be valuable for the intervention of NAFLD.

## Data Availability

All data generated or analyzed in this study are available from the corresponding author for the reasonable request.

## Abbreviations

ALT: Alanine aminotransferase
Ara: Arabinose
AST: Aspartate aminotransferase
CLP: Chaetomorpha linum polysaccharide
CPT-1: Carnitine palmityl transferase-1
FBG: Fasting blood glucose
Gal: Galactose
Glu: Glucose
GlcA: Glucuronic acid
HDL: High-density lipoprotein
H&E: Hematoxylin and eosin
HFD: High fat diet
LDL: Low-density lipoprotein
MCAD: Mid-chain acyl-coA dehydrogenase
NAFLD: Nonalcoholic fatty liver disease
OGTT: Oral glucose tolerance test
PMP-HPLC: 1 Phenyl 3-methyl 5-pyrazolone high performance liquid chromatography
PPARα: Peroxisome proliferator-activated receptor α
qRT-PCR: Quantitative real-time polymerase chain reaction
Rha: Rhamnose
SE: Standard error
TC: Total cholesterol
TG: Triglyceride
